# A Numerical Study to Compare Stimulations by Intraoperative Microelectrodes and Chronic Macroelectrodes in the DBS Technique

**DOI:** 10.1155/2013/262739

**Published:** 2013-10-07

**Authors:** A. Paffi, F. Apollonio, M. G. Puxeddu, M. Parazzini, G. d'Inzeo, P. Ravazzani, M. Liberti

**Affiliations:** ^1^Department of Information Engineering, Electronics and Telecommunication, Sapienza University of Rome, 00184 Rome, Italy; ^2^Italian Inter-University Center for the Study of Electromagnetic Fields and Biological Systems (ICEmB), 16145 Genova, Italy; ^3^CNR Consiglio Nazionale delle Ricerche, Istituto di Elettronica e di Ingegneria dell'Informazione e delle Telecomunicazioni IEIIT, 20133 Milano, Italy

## Abstract

Deep brain stimulation is a clinical technique for the treatment of parkinson's disease based on the electric stimulation, through an implanted electrode, of specific basal ganglia in the brain. To identify the correct target of stimulation and to choose the optimal parameters for the stimulating signal, intraoperative microelectrodes are generally used. However, when they are replaced with the chronic macroelectrode, the effect of the stimulation is often very different. Here, we used numerical simulations to predict the stimulation of neuronal fibers induced by microelectrodes and macroelectrodes placed in different positions with respect to each other. Results indicate that comparable stimulations can be obtained if the chronic macroelectrode is correctly positioned with the same electric center of the intraoperative microelectrode. Otherwise, some groups of fibers may experience a completely different electric stimulation.

## 1. Introduction

Deep brain stimulation (DBS) is a successful technique in reducing symptoms of several neurological disorders, particularly effective in the treatment of advanced Parkinson's disease (PD) [1, 2]. It is based on the stimulation, through an implanted electrode, of the basal ganglia in the brain using a train of electric biphasic pulses with a main frequency between 120 and 180 Hz. Although the health-related quality of the life of the patients is actually improved by this technique, the precise mechanism of DBS functioning remains still unclear [1]. Similar to other techniques used in the brain stimulation, such as the low-level magnetic stimulation [3], the choice of both the brain target and the stimulation signal is based more on empirical observations than on the precise knowledge of the mechanisms of action on brain structures.

Clinic experience has evidenced that DBS maximizes the beneficial effects on PD motor symptoms if the electric stimulation is localized in the subthalamic nucleus (STN). The STN is a small lens-shaped nucleus responsible for body movements and coordination. The main connections of the STN are with the globus pallidus (Gp), which carries output from caudate nucleus (Cd) and putamen (Pt) to the thalamus (Th) [4–6]. All these structures form the anatomical nuclei of the basal ganglia and are located deep within the cerebral hemispheres. Many papers have reported clinical results supporting the hypothesis that STN represents the most suitable target for the DBS treatment of PD [7–11].

To identify this target nucleus, during stereotactic surgery, intraoperative microelectrode recordings of neuronal activities are performed; in the meanwhile, the same microelectrode is used for the microstimulation of neuronal tissue to optimize the stimulation parameters, in terms of amplitude, frequency, and pulse width of the electric signal [12]. 

After this step, the microelectrode is replaced by the chronic one (macroelectrode) fixed in the same location. Nevertheless, the stimulation with a macro- or a microelectrode does not always induce similar clinical effects [9] consisting in minimizing the symptoms while reducing the side effects. Due to the great difference in size between macroelectrode and microelectrode (almost two orders of magnitude), these discrepancies could be attributable to a more localized stimulation obtained with the microelectrode but also to an uncorrected positioning of the chronic macroelectrode. Indeed, the concept of “same location” is not unambiguous; for the macroelectrode to have the same location of the microelectrode, it is sufficient that this latter is included in the volume occupied by the macroelectrode. 

To understand the specific mechanisms underlying the aforementioned discrepancies and to predict the fiber responses to different kinds of stimulation, an integrated approach should be followed [13, 14], coupling dosimetric models, whose outputs are the field distribution inside the biological target, with biophysical ones to evaluate the response of the exposed structures to the field [15].

In this context, the first step is the evaluation, through accurate dosimetric models, of the electric potential (*V*) and the activating function (AF) generated inside the neuroanatomical nuclei by the electric stimulation. At this stage, the AF [16], that is, the second space derivative of *V* along the fiber direction, is a particularly interesting parameter since it can furnish a first qualitative evaluation of the excitation or inhibition of neuronal fibers.

In previous works, the authors, on the bases of NMR images and stereotactic Atlas, have developed accurate 2D [17] and 3D [18] dosimetric models. In those works, geometric parameters of the analysis domain and the ground positioning on the domain boundary were optimized as the best compromise between the computational effort and the solution accuracy. 

The 3D model was numerically solved in [19] under the stimulation of the macro- or microelectrode trying to explain the different behaviors observed. In that study, the microelectrode and the macroelectrode were placed so that the extremities of the leads, inside the STN, were coincident. Preliminary results showed that the AF, calculated along a single line representative of a fiber, had opposite behaviors depending on the kind of stimulation. Such an unexpected result was hypothesized to be due to the relative position of the fiber with respect to the active contacts of the macroelectrode or the microelectrode. 

In this study, this hypothesis is in depth investigated, comparing the stimulation induced on 12 different fibers by the macroelectrode with those induced by the microelectrode placed in two different positions. The purpose is to accurately characterize the stimulation of the two kinds of electrodes in terms of the values of *V* and AF on different groups of fibers. 

The final objective is to contribute to clarify when the two stimulations are equivalent and which are the practical protocols to follow.

## 2. Models and Methods

### 2.1. Dosimetric Model

Due to the low frequency content of the stimulating signal (up to a few kHz), the minimum wavelength is much higher than the ganglia size (order of some cm) and the problem can be treated as a quasi-static one [11, 20]. Thus, the Laplace equation is solved using the software package Comsol Multiphysics v.3.4 (Comsol Inc.) based on finite element methods [20]. 

**Figure 1:**
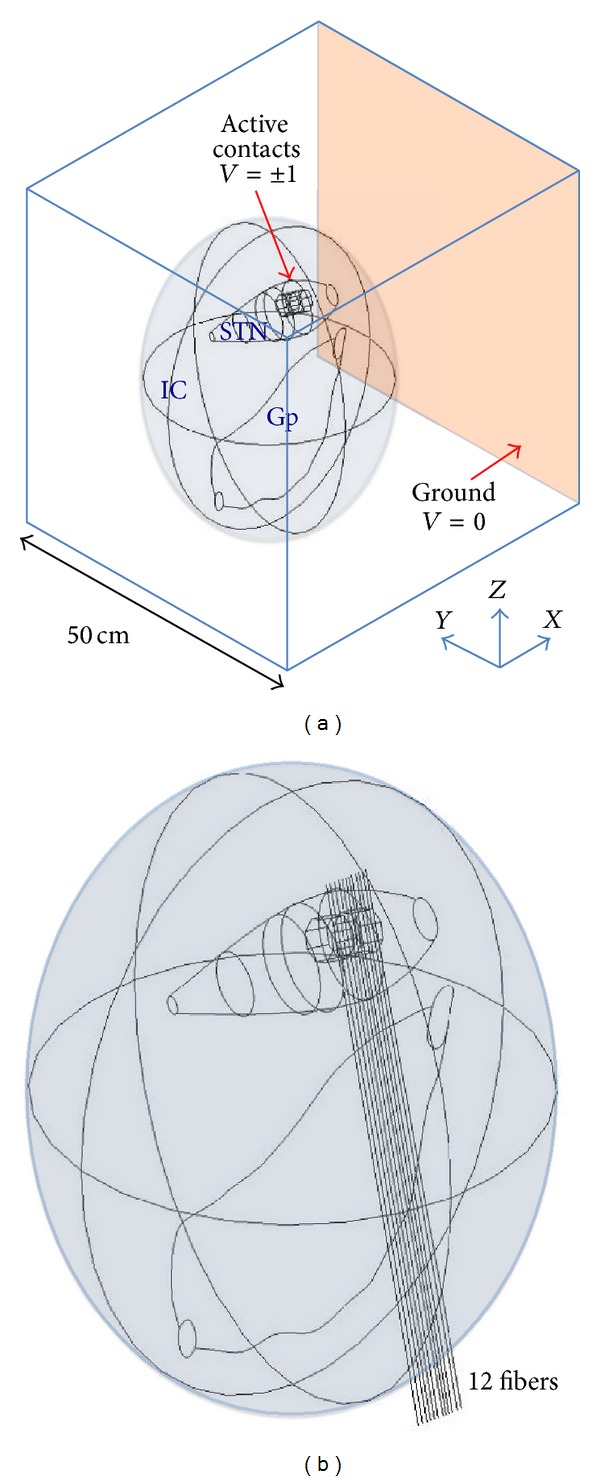
(a) 3D dosimetric model; (b) set of 12 lines representative of the neuronal fibers connecting the STN to the Gp and passing through the IC.

The used 3D model, obtained from clinician MRI data, is reported in Figure 1(a). The model of the basal ganglia encompasses STN, Gp, and the internal capsule (IC). STN and Gp are particularly important since neural activity between these anatomical nuclei [10, 11] is impaired in PD; IC is a white matter region surrounding basal nuclei, composed of bundles of long fibers which link STN and Gp; its anisotropic properties are due to the fibers direction [21]. The model, following the approach proposed in [22], takes into account both isotropic and anisotropic properties of the tissues, as described in [18], and in particular Gp and STN are modeled as isotropic grey matter (*σ* = 0.2 S/m). The IC has been modeled as a uniaxially anisotropic medium [17] (*σ*_*yy*_ = *σ*_*xx*_ = 0.1 S/m, *σ*_*zz*_ = 1 S/m) of spheroidal shape with the main axis parallel to the fiber direction (*z*-axes, Figure 1(a)) and added around the two anatomical nuclei into the 3D volume conductor modeled as a cubic box (Figure 1(a)), 50 cm of side, filled with an isotropic medium representative of the brain tissue (*σ* = 0.09 S/m). 

**Figure 2:**
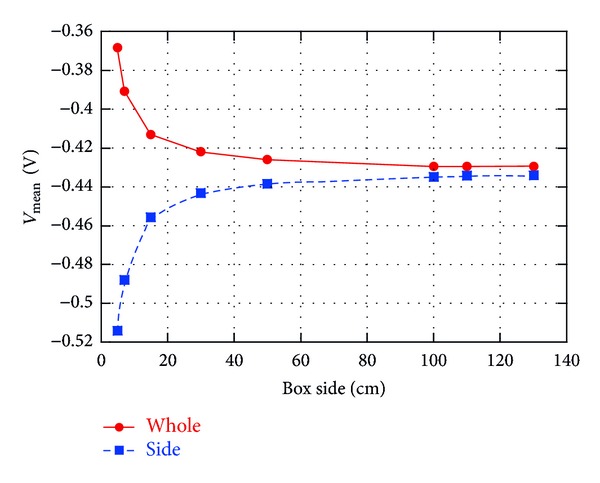
Variations of the electric potential, averaged over the STN volume, with the dimension of the box, representing the analysis domain, for a monopolar stimulation (stimulating voltage: −1 V) and two boundary conditions: ground on the whole lateral surface (whole) and on one face (side).

The value of 50 cm was evaluated in [18] by considering the variations with the box size of *V*, averaged over the STN volume (*V*_mean_), for a monopolar stimulation (one active contact of the electrode set to −1 V).  Figure 2, whose data are taken from Figure 3 of [18], shows the estimated *V*_mean_ versus the box size, for two different boundary conditions, that is, with the ground on the whole lateral surface (whole) or on one face (side). In the “whole” ground condition, *V*_mean_ decreases and tends to saturate for increasing box dimensions; in particular, changes are less than 1% if the box side is at least 50 cm. Similar percentages are obtained in the “side” condition, even if *V*_mean_ increases with the box dimensions. Therefore, a box 50 × 50 × 50 cm^3^ has been chosen for numerical simulations as the best compromise between computational effort and solution accuracy. Such a choice seems also the most appropriate from an anatomical point of view being 25 cm a reasonable “average” distance between the electrode in the center of the brain and the case of the stimulator in the subclavicular region.

### 2.2. Stimulation

The considered stimulating leads are the Medtronic “3389” [23] as the chronic one, and the commercial quadruple microelectrode (FHC Inc.) as the intraoperative electrode used during the surgical operation. Only two active contacts of both stimulating macro- and microelectrodes have been taken into account and modeled as platinum contacts (*σ* = 8.6 · 10^6^ S/m) with octagonal section. With respect to a circular section, an octagonal modeling of the active contacts permits a simpler discretization of the surfaces, thus minimizing numerical errors. The first electrode (macro) has an external diameter of 1.27 mm, height of 1.5 mm, and interdistance of 0.5 mm; the second one (micro) has external diameter, height, and interdistance of 35 *μ*m. In this work, the bipolar configuration for the electrodes has been considered since it is the most used during the surgical procedure to optimize the correct position for the chronic DBS stimulating electrode of STN. For both electrodes, *Contact 0 *was set as the negative contact (*V* = −1 V) and *Contact 1 *as the positive one (*V* = +1 V) according to the specifications reported in [1, 23]. Since the active surface of the microelectrode is much smaller than the macroelectrode one (1000 times smaller), to have the same current density injected in the tissue, in order to properly compare data from both stimulations, the *V* of the microelectrode contacts has been multiplied by the scale factor of 1000. The ground has been placed on one face of the cube (Figure 1(a)), in the so-called “side” configuration [18]. As evident from Figure 2, for a box of at least 50 cm of side, the ground positioning is not significant (variations in *V*_mean_ below 3%); however, the “side” configuration is more realistic from an anatomical point of view, since the ground is placed on the case of the implanted pulse generator, thus laterally with respect to the electrode.

**Figure 3:**
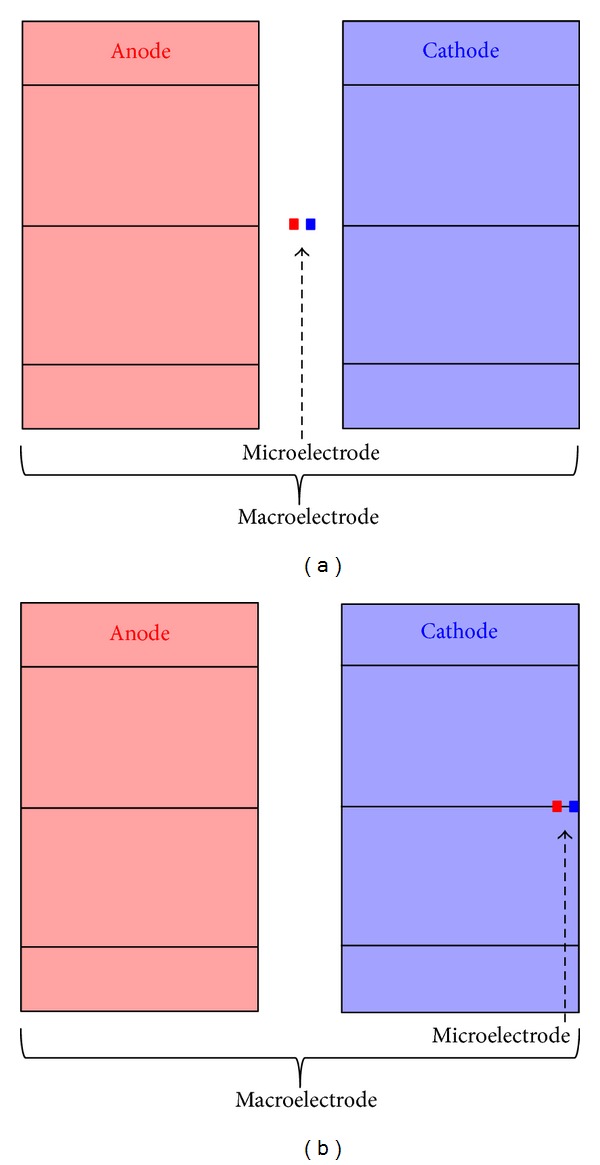
Relative positions of the microelectrode with respect to the macroelectrode one (not in scale). (a) Position 1, with the same electric center; (b) position 2, with the external surfaces of the cathodes coincident.

According to the clinical practice, both contacts of macroelectrode are placed inside the STN (Figure 1). The microelectrode is placed in two different positions with respect to the macroelectrode: with the same electrical center (position 1), with the external surfaces of the cathodes coincident (position 2) (Figure 3).

The solution was obtained using a tetrahedral mesh. Due to the great difference in dimensions of different subdomains, the mesh density was manually set in a not uniform way. Quadrupling the density of the mesh, variations in distributions of the electric quantities remained below 1%.

In order to evaluate the responses of the excitable tissue in three different stimulation conditions: (i) using the macroelectrode, (ii) using the microelectrode in position 1, and (iii) using the microelectrode in position 2, the electric potential *V* and the AF have been calculated on a set of 12 lines representative of the neuronal fibers that connect the STN to the Gp passing through the IC (Figure 1(b)). The lines form an angle of 10° with the *z* axis and pass at a distance from the active contacts of the macroelectrode that varies between 2 and 2.5 mm.

### 2.3. Observables

Results of the simulations, conducted with the two types of electrode, are based on the evaluation of the distribution of *V* and AF along the 12 lines (Figure 1(b)), representative of fibers direction, passing through the Gp and the STN. The AF is the second derivative of the extracellular potential along the axis of a fiber [16]. On the basis of the classical cable theory, long and straight nervous fibers may be activated or inhibited depending on whether AF is positive or negative [16]. The threshold value for the AF, able to induce activation or inhibition of the fiber, depends on the specific features of the fiber and may be defined only coupling the dosimetric analysis with neuronal modeling [24]. Therefore, if this threshold is not known, the sign of the AF can be used to qualitatively estimate the regions of depolarization and hyperpolarization generated along the neuronal fibers by the stimulating electrode [16].

## 3. Results

**Figure 4:**
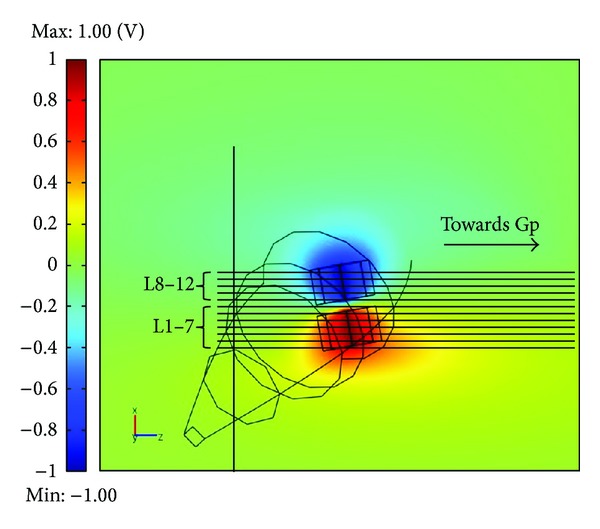
Distribution of the electric potential *V* induced by the macroelectrode on a plane parallel to *xz*. The projections of the 12 lines on the plane are highlighted. Among them, two groups have been identified: L1–7, passing closer to the anode; L8–12, passing closer to the cathode.

As a first result, Figure 4 reports that the *V* distribution is induced by the macroelectrode on a plane parallel to the *xz* one. As evident, the stimulation is mainly confined within the STN, but a spread into the IC is observable, especially in correspondence to the anode, along the anisotropy, that is, the *z* axis. 

In Figure 4 the projections of the 12 considered lines are highlighted and divided into two groups, depending on the position of each line with respect to the anode and the cathode of the macroelectrode.

**Figure 5:**
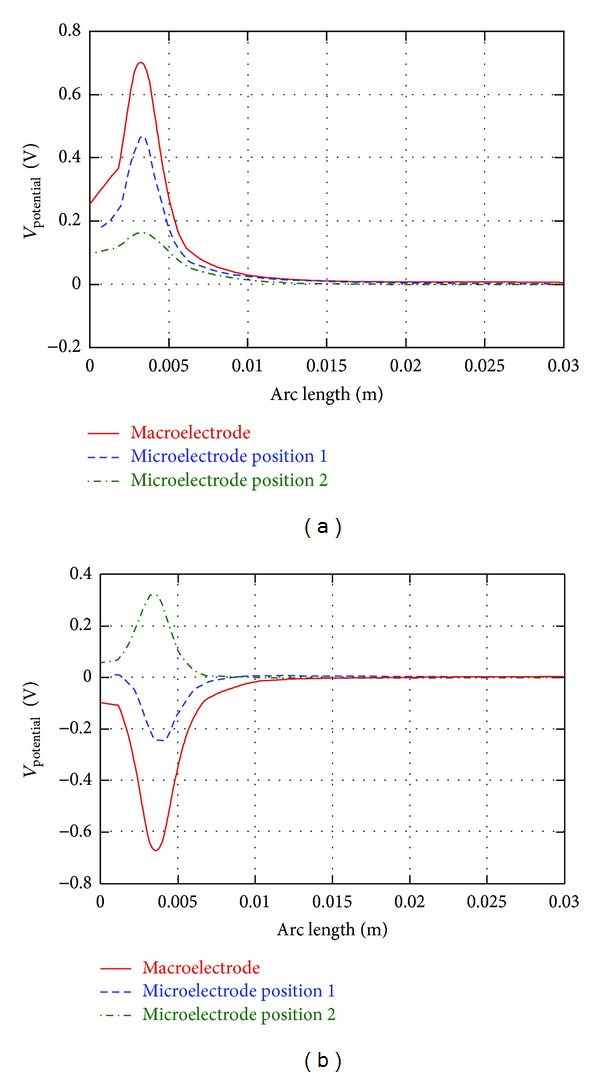
(a) Electric potential *V* along the 3rd line (L3) under the stimulations with the macroelectrode, the microelectrode in position 1, and the microelectrode in position 2; (b) electric potential *V* along the 11th line (L11) under the stimulations with the macroelectrode, the microelectrode in position 1, and the microelectrode in position 2.

The values of *V* and AF along all the 12 lines have been calculated for the three considered stimulation conditions: macroelectrode, microelectrode in position 1 and microelectrode in position 2 (Figure 3). In Figure 5, the electric potential *V* is reported along the 3rd line (Figure 5(a)), belonging to the group L1–7, and the 11th line (Figure 5(b)), belonging to the group L8–12, for the three kinds of stimulation. Looking at Figure 5(a), one can see that *V* is always positive, independently on the kind of stimulation; indeed, due to its position with respect to the active contacts, the 3rd fiber (L3) is always affected by the anodic stimulation. Conversely, the maximum values reached by *V* along the line are different and essentially depend on the distance between the fiber and the anode. Therefore, although the electric potential on the surfaces of the microelectrode anode is 1000 times higher than on the macroelectrode one (see Section 2.2), the higher distance of the line from the anode makes the values of *V* lower with the microelectrode stimulation, especially when it is placed laterally (position 2, Figure 3). Similar behaviors are obtained for all the lines of the group L1–7. 

Figure 5(b) reports the same observable on the 11th line (L11) of the group L8–12. In this case, *V* is negative when using the macroelectrode and the microelectrode in position 1 (Figure 3(a)), and positive with the microelectrode in position 2 (Figure 3(b)). Again, this is due to the fact that, for all the positions, apart for the latter one, the fiber is closer to the cathode; hence the potential is negative. The electric potential *V* exhibits the same behavior for all the fibers of group L8–12.

**Figure 6:**
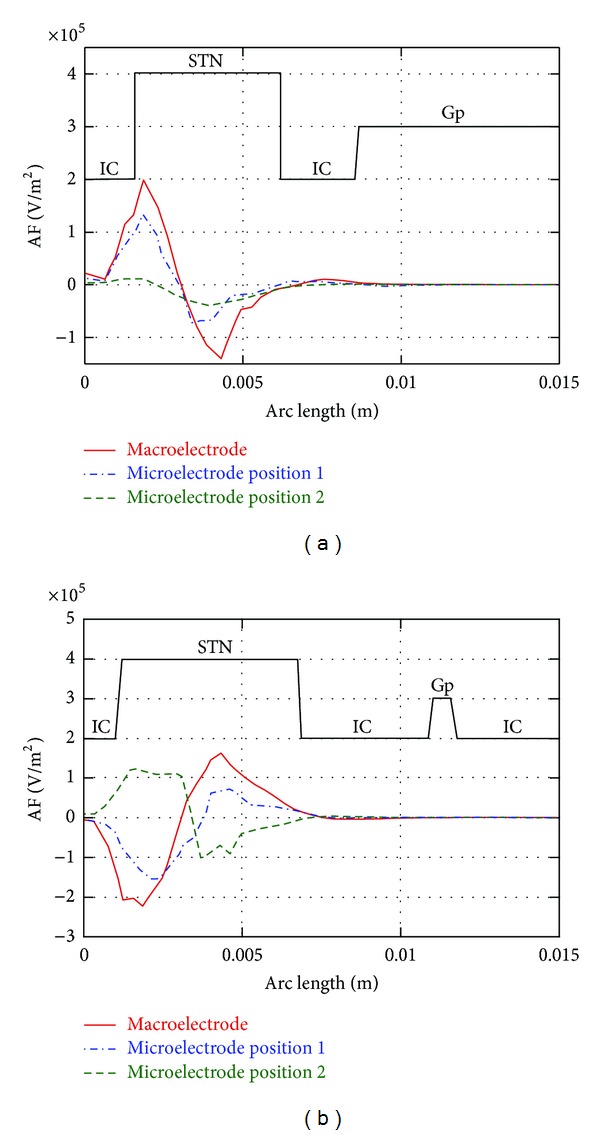
(a) AF along the 4th line (L4) under the stimulations with the macroelectrode, the microelectrode in position 1, and the microelectrode in position 2; (b) AF along the 12th line (L12) under the stimulations with the macroelectrode, the microelectrode in position 1, and the microelectrode in position 2. The black traces represent the brain regions crossed by the lines.

Moving to the AF, Figure 6 shows its behavior along L4 (Figure 6(a)) and L12 (Figure 6(b)), and for the three kinds of stimulations. In all cases, the AFs show a biphasic trend. The two phases along the line indicate that the same fiber may be excited in the region where the AF is positive and inhibited where the AF is negative. This is in agreement with theoretical and experimental results [25] reporting activation or inhibition of the fibers during the DBS stimulation, depending on whether the electrophysiological recordings were made on the soma or on the axon.

Therefore, it is important to define where the two regions (positive AF and negative AF) are placed inside the neuroanatomical target. To do that, in Figure 6, the “index of domain” (black line) is plotted together with the AF. This is a Comsol function that assigns different conventional numbers to each domain (STN, Gp, IC, and brain) crossed by the line. As evident from Figure 6(a), all kinds of stimulations induce on L4 an AF passing from positive to negative values. The AFs induced by macroelectrode and microelectrode in position 1 are comparable in intensity, whereas microelectrode in position 2 induces peaks of the AF much less intense, due to the higher distance of the fiber (L4) from the active contacts. However, as explained in Section 2.3, the intensity of the AF cannot be related straightforward to the intensity of the stimulation of the neuronal tissue [24], as, conversely, is for its sign.

As for the L12 (Figure 6(b)), passing close to the cathode of the macroelectrode, the phase of the AF passes from negative values to positive ones for stimulations with the macroelectrode and with the microelectrode in position 1 whereas when the microelectrode is placed in position 2, the behavior of the AF is opposite, with similar absolute values.

This result, obtained even for the other lines of the same group, is particularly significant because it demonstrates that the response of the fiber may be completely different between macroelectrode and microelectrode stimulation, depending on whether the two kinds of electrodes are “centered” (data coincide for all the fiber lines) or “lateral” (data can be very different depending on the group of the fiber lines examined).

Regarding the stimulation spread, Figure 6 shows that it is mainly concentrated in the STN, where the soma of the neuronal fibers are present, but it extents also to the IC, thus affecting the axons.

A summary of the signs assumed by the AF along all the 12 lines and for the three stimulations is reported in Table 1.

It is evident that discrepancies between macro- and microstimulation are present in the group of fibers L8–12 depending on whether the microelectrode is placed with the same electric center of the macroelectrode (position 1) or lateral (position 2).

The macroelectrodes and the microelectrodes, if applied with the electric center coincident (position 1), induce in the brain tissue electric stimulations with the same trends of *V* and AF for all the 12 fibers. In general, the trends depend on the geometry of the problem, namely, on the position of the active contacts with respect to the fiber under test.

Conversely, when the microelectrodes and the macroelectrodes coincide laterally (position 2), the two kinds of stimulation are completely opposite.

## 4. Conclusions

Results of this study show that, as already hypothesized by the authors, different trends of the observable functions may be obtained between micro- and macrostimulation, depending on the fiber under examination and, in general, on the portion of the tissue to be stimulated.

If we want the chronic electrode to produce the same effects of intraoperative microelectrode, having identified with this the optimal parameters of stimulation, then it will be necessary to place the macroelectrode so that the electric center coincides with the electric center of the microelectrode. In this way it will give rise to the same trends of *V* and AF along all the fibers connecting the two nuclei STN and Gp.

To obtain a quantitative evaluation of the response of fibers, a further development of this work will be the coupling of this dosimetric model with biophysical models of neuron and networks, as those used in [15, 26, 27]. 

Since in the biophysical models the transmembrane potential has to be inserted as the input parameter of the stimulation, this quantity has to be rigorously evaluated. This can be done using microdosimetric techniques for single cell [28–33], which allow us to calculate the electric quantities at the microscopic level of the single cell and among them precisely the membrane potential. In this way a link between dosimetry at the macroscopic level of tissues and neuronal models will be performed opening the way to an approach based on a multilevel methodology, unavoidable when the final aim is to identify practical protocols to be followed in clinical practice.

## Figures and Tables

**Table 1:** Signs of AF along the 12 fibers for the three stimulations: macroelectrode and microelectrode in positions 1 and 2.

	L1	L2	L3	L4	L5	L6	L7	L8	L9	L10	L11	L12
Macro	+ −	+ −	+ −	+ −	+ −	+ −	+ −	− +	− +	− +	− +	− +
Micro (position 1)	+ −	+ −	+ −	+ −	+ −	+ −	+ −	− +	− +	− +	− +	− +
Micro (position 2)	+ −	+ −	+ −	+ −	+ −	+ −	+ −	+ −	+ −	+ −	+ −	+ −
